# Draft Genome Sequence of the Type Strain *Aeromonas salmonicida* subsp. *salmonicida* ATCC 33658

**DOI:** 10.1128/genomeA.01064-17

**Published:** 2017-10-05

**Authors:** Katherinne Valderrama, Manuel Soto-Davila, Javier Santander

**Affiliations:** Marine Microbial Pathogenesis and Vaccinology Laboratory, Department of Ocean Sciences, Memorial University of Newfoundland, St. John's, Canada

## Abstract

Here, we report the draft genome sequence of the type strain *Aeromonas salmonicida* subsp. *salmonicida* ATCC 33658 isolated from *Salmo salar*. The size of the genome is 4,728,143 bp with a G+C content of 58.5%. The *A. salmonicida* subsp. *salmonicida* ATCC 33658 genome lacks essential virulence genes that were likely lost during genomic rearrangements.

## GENOME ANNOUNCEMENT

First described in the 19th century, *Aeromonas salmonicida* subsp. *salmonicida* is one of the oldest known fish pathogens. *A. salmonicida* subsp. *salmonicida* is an important pathogen due to its nearly worldwide distribution, broad host range, and potentially devastating impacts on wild and farm fish. *A. salmonicida* subsp. *salmonicida*, a Gram-negative, nonmotile gammaproteobacteria, is the etiological agent of the so-called “typical” furunculosis. *A. salmonicida* subsp. *salmonicida* ATCC 33658 (1), isolated from Atlantic salmon (*Salmo salar*), has been utilized as the type strain for phylogenetic (2), detection (3, 4), and pathogenesis studies (5, 6). Additionally, *A. salmonicida* subsp. *salmonicida* ATCC 33658 is the type strain for antimicrobial susceptibility testing of aquatic bacteria (7). Although it has been recognized that this strain has lost several virulence genes (6, 8), its genome remains uncharacterized. Here, we report the draft genome sequence of *A. salmonicida* subsp. *salmonicida* ATCC 33658, a nonvirulent strain that lacks the A-layer and type III secretion-related genes.

*A. salmonicida* subsp. *salmonicida* ATCC 33658 was routinely grown in Trypticase soy broth (TSA) (Difco) with aeration (180 rpm) at 15°C. The genomic DNA was extracted according to Wilson (9) and purified using silica (10). Sequencing was performed using the Illumina MiSeq next-generation sequencing platform (Universidad Mayor, Center for Genomics and Bioinformatics, Huechuraba, Chile) and paired-end libraries. Low-quality sequences were examined by FastQC version 0.10.1 (11). The sequences were trimmed and assembled using the CLC Genomics Workbench version 10.1.1 (Qiagen) *de novo* tool, which resulted in 119 contigs over 1 kb with an *N*_50_ of 89,474 bp. The total length of the draft genome of *A. salmonicida* subsp. *salmonicida* ATCC 33658 strain J208 was 4,728,143 bp with a G+C content of 58.5%.

The assembled sequences were annotated by the NCBI Prokaryotic Genome Annotation Pipeline (https://www.ncbi.nlm.nih.gov/genome/annotation_prok). The tRNAs were detected by tRNA scan-SE version 1.3 (12) and the rRNAs with RNAmmer (13). A total of 4,273 coding sequences, 208 pseudogenes, 5 complete rRNA operons (5S-16S-23S), 81 tRNAs, and 4 noncoding RNAs were predicted by the pipeline.

The S-layer of *A. salmonicida* subsp. *salmonicida* is an essential virulence factor that allows *A. salmonicida* subsp. *salmonicida* to resist the complement and ultimately infects and kills the fish host. (14–16). The S-layer is an array formed by the VapA protein (17), whose encoding gene is susceptible to inactivation by thermal inducible insertion elements (IS) (18, 19). We found that the *vapA* gene is truncated, likely by an IS during previous culture and passages. As a consequence, *A. salmonicida* subsp. *salmonicida* ATCC 33658 shows an A^−^ phenotype and no expression of the VapA protein (20). Also, as described previously (8), *A. salmonicida* subsp. *salmonicida* ATCC 33658 lacks type III secretion-related genes, like *acsV* and *ascR*.

The resistome was identified using the Comprehensive Antibiotic Resistance Database (21). MCR-3 related to polymyxin resistance (contig 119, 224 to 1,849 bp) and carbapenem-hydrolyzing metallo-beta-lactamase (*cphA5*) related to beta-lactam resistance (contig 83, 10,993 to 11,754 bp) genes were identified. *A. salmonicida* subsp. *salmonicida* ATCC 33658 is susceptible to ampicillin and polymyxin, suggesting that these genes are inactive or require activation.

### Accession number(s).

This whole-genome shotgun project (BioProject PRJNA310296) has been deposited at DDBJ/EMBL/GenBank under accession number LSGW00000000. The version described here is the first version, LSGW01000000.

## References

[1] Griffin PJ, Snieszko SF, Friddle SB 1953 Pigment formation by *Bacterium salmonicida*. J Bacteriol 65:652–659. 13069437 10.1128/jb.65.6.652-659.1953 PMC169594

[2] Martínez-Murcia AJ, Soler L, Saavedra MJ, Chacón MR, Guarro J, Stackebrandt E, Figueras MJ 2005 Phenotypic, genotypic, and phylogenetic discrepancies to differentiate *Aeromonas salmonicida* from *Aeromonas bestiarum*. Int Microbiol 8:259–269. 16562378

[3] Keeling SE, Brosnahan CL, Johnston C, Wallis R, Gudkovs N, McDonald WL 2013 Development and validation of a real-time PCR assay for the detection of *Aeromonas salmonicida*. J Fish Dis 36:495–503. doi:10.1111/jfd.12014. 23121198

[4] Sugita H, Nakamura T, Tanaka K, Deguchi Y 1994 Identification of *Aeromonas* species isolated from freshwater fish with the microplate hybridization method. Appl Environ Microbiol 60:3036–3038. 16349363 10.1128/aem.60.8.3036-3038.1994 PMC201763

[5] Stuber K, Burr SE, Braun M, Wahli T, Frey J 2003 Type III secretion genes in *Aeromonas salmonicida* subsp. *salmonicida* are located on a large thermolabile virulence plasmid. J Clin Microbiol 41:3854–3856. doi:10.1128/JCM.41.8.3854-3856.2003. 12904401 PMC179850

[6] Braun M, Stuber K, Schlatter Y, Wahli T, Kuhnert P, Frey J 2002 Characterization of an ADP-ribosyltransferase toxin (AexT) from *Aeromonas salmonicida* subsp. *salmonicida*. J Bacteriol 184:1851–1858. doi:10.1128/JB.184.7.1851-1858.2002. 11889090 PMC134929

[7] Miller RA, Walker RD, Baya A, Clemens K, Coles M, Hawke JP, Henricson BE, Hsu HM, Mathers JJ, Oaks JL, Papapetropoulou M, Reimschuessel R 2003 Antimicrobial susceptibility testing of aquatic bacteria: quality control disk diffusion ranges for *Escherichia coli* ATCC 25922 and *Aeromonas salmonicida* subsp. *salmonicida* ATCC 33658 at 22 and 28°C. J Clin Microbiol 41:4318–4323. doi:10.1128/JCM.41.9.4318-4323.2003. 12958263 PMC193829

[8] Burr SE, Stuber K, Frey J 2003 The ADP-ribosylating toxin, AexT, from *Aeromonas salmonicida* subsp. *salmonicida* is translocated via a type III secretion pathway. J Bacteriol 185:6583–6591. doi:10.1128/JB.185.22.6583-6591.2003. 14594831 PMC262089

[9] Wilson K 2001 Preparation of genomic DNA from bacteria. Curr Protoc Mol Biol Chapter2:Unit 2.4. doi:10.1002/0471142727.mb0204s56. 18265184

[10] Boom R, Sol C, Beld M, Weel J, Goudsmit J, Wertheim-van Dillen P 1999 Improved silica-guanidiniumthiocyanate DNA isolation procedure based on selective binding of bovine alpha-casein to silica particles. J Clin Microbiol 37:615–619. 9986822 10.1128/jcm.37.3.615-619.1999 PMC84491

[11] Andrews S 2010 FastQC: a quality control tool for high throughput sequence data. http://www.bioinformatics.babraham.ac.uk/projects/fastqc.

[12] Lowe TM, Eddy SR 1997 tRNAscan-SE: a program for improved detection of transfer RNA genes in genomic sequence. Nucleic Acids Res 25:955–964. 9023104 10.1093/nar/25.5.955 PMC146525

[13] Lagesen K, Hallin P, Rødland EA, Staerfeldt HH, Rognes T, Ussery DW 2007 RNAmmer: consistent and rapid annotation of ribosomal RNA genes. Nucleic Acids Res 35:3100–3108. doi:10.1093/nar/gkm160. 17452365 PMC1888812

[14] Ishiguro EE, Kay WW, Ainsworth T, Chamberlain JB, Austen RA, Buckley JT, Trust TJ 1981 Loss of virulence during culture of *Aeromonas salmonicida* at high temperature. J Bacteriol 148:333–340. 7287625 10.1128/jb.148.1.333-340.1981 PMC216197

[15] Kay WW, Trust TJ 1991 Form and functions of the regular surface array (S-layer) of *Aeromonas salmonicida*. Experientia 47:412–414. 2044685

[16] Garduño RA, Moore AR, Olivier G, Lizama AL, Garduño E, Kay WW 2000 Host cell invasion and intracellular residence by *Aeromonas salmonicida*: role of the S-layer. Can J Microbiol 46:660–668. doi:10.1139/w00-034. 10932360

[17] Chu S, Cavaignac S, Feutrier J, Phipps BM, Kostrzynska M, Kay WW, Trust TJ 1991 Structure of the tetragonal surface virulence array protein and gene of *Aeromonas salmonicida*. J Biol Chem 266:15258–15265. 1869553

[18] Ishiguro EE, Ainsworth T, Trust TJ, Kay WW 1985 Congo red agar, a differential medium for *Aeromonas salmonicida*, detects the presence of the cell surface protein array involved in virulence. J Bacteriol 164:1233–1237. 3934141 10.1128/jb.164.3.1233-1237.1985 PMC219320

[19] Gustafson CE, Chu SJ, Trust TJ 1994 Mutagenesis of the paracrystalline surface protein array of *Aeromonas salmonicida* by endogenous insertion elements. J Mol Biol 237:452–463. doi:10.1006/jmbi.1994.1247. 8151705

[20] Valderrama K, Saravia M, Santander J 2017 Phenotype of *Aeromonas salmonicida* sp. *salmonicida* cyclic adenosine 3′,5′-monophosphate receptor protein (Crp) mutants and its virulence in rainbow trout (*Oncorhynchus mykiss*). J Fish Dis 7:1–8. doi:10.1111/jfd.12658. 28548689

[21] McArthur AG, Waglechner N, Nizam F, Yan A, Azad MA, Baylay AJ, Bhullar K, Canova MJ, De Pascale G, Ejim L, Kalan L, King AM, Koteva K, Morar M, Mulvey MR, O’Brien JS, Pawlowski AC, Piddock LJ, Spanogiannopoulos P, Sutherland AD, Tang I, Taylor PL, Thaker M, Wang W, Yan M, Yu T, Wright GD 2013 The comprehensive antibiotic resistance database. Antimicrob Agents Chemother 57:3348–3357. doi:10.1128/AAC.00419-13. 23650175 PMC3697360

